# From waste to health: sustainable exploitation of grape pomace seed extract to manufacture antioxidant, regenerative and prebiotic nanovesicles within circular economy

**DOI:** 10.1038/s41598-020-71191-8

**Published:** 2020-08-25

**Authors:** Maria Letizia Manca, Eleonora Casula, Francesca Marongiu, Gianluigi Bacchetta, Giorgia Sarais, Marco Zaru, Elvira Escribano-Ferrer, José Esteban Peris, Iris Usach, Sara Fais, Alessandra Scano, Germano Orrù, Richard G. Maroun, Anna Maria Fadda, Maria Manconi

**Affiliations:** 1 Section of Pharmaceutical Sciences, Department of Life and Environmental Sciences, University of Cagliari, Via Ospedale 72, 09124 Cagliari, Italy; 2 Icnoderm Srl, Sardegna Ricerche Ed. 5, Pula, 09010 Cagliari, Italy; 3 Biopharmaceutics and Pharmacokinetics Unit, Institute for Nanoscience and Nanotechnology, University of Barcelona, Barcelona, Spain; 4 Department of Pharmacy and Pharmaceutical Technology and Parasitology, University of Valencia, Burjassot, 46100 Valencia, Spain; 5 Department of Surgical Science, Molecular Biology Service Lab (MBS), University of Cagliari, Via Ospedale 40, 09124 Cagliari, Italy; 6 Centre d’Analyses et de Recherche, UR GPF, Laboratoire CTA, Faculté Des Sciences, Université Saint-Joseph, B.P. 11-514 Riad El Solh, Beirut, 1107 2050 Lebanon

**Keywords:** Health care, Nanoscience and technology

## Abstract

Pomace seed extract loaded vesicles were prepared as promising technological and green solution to exploit agri-food wastes and by-products, and develop high value-added products for human health. An antioxidant extract rich in bioactive compounds (epicatechins, catechin, gallic acid, quercetin and procynidins) was obtained from the seeds isolated from the pomace of Cannonau red grape cultivar. The extract was incorporated into phospholipid vesicles ad hoc formulated for intestinal delivery, by combining them, for the first time, whit a maltodextrin (Glucidex). Glucidex-transfersomes, glucidex-hyalurosomes and glucidex-hyalutransferomes were prepared, characterized and tested. Glucidex-liposomes were used as reference. All vesicles were small in size (~ 150 nm), homogeneously dispersed and negatively charged. Glucidex-transfersomes and especially glucidex-hyalutransfersomes disclosed an unexpected resistance to acidic pH and high ionic strength, as they maintained their physico-chemical properties (size and size distribution) after dilution at pH 1.2 simulating the harsh gastric conditions. Vesicles were highly biocompatible and able to counteract the oxidative damages induced in Caco-2 cells by using hydrogen peroxide. Moreover, they promoted the formation of *Lactobacillus reuteri* biofilm acting as prebiotic formulation. Overall results suggest the potential of glucidex-hyalutransfersomes as food supplements for the treatment of intestinal disorders.

## Introduction

The circular economy involves the creation of a closed-loop ecosystem for effective consumption and utilization of resources by the adoption of reduce, reuse, and recycle paradigms^1^. According to this paradigm, reducing the generation of wastes through their recycle and reuse as well as the sustainable management and efficient use of natural resources are the main challenges for the modern circular economy. A transition towards a restorative and regenerative economic cycle based on more sustainable practices and reduced environmental impacts of production and consumption is required. Several studies have been performed to evaluate the potential strategies aimed at converting food wastes and by-products into value-added products, especially food nutrients or supplements^2,3^. This sector has gained great interest in recent years due to the increasing attention to human health in response to the incorrect *modus vivendi* of modern people, which is characterized by stress, lack of sleep, intake of junk food, alcohol, smoke and drugs. The incorrect life style and the intake of external unhealthy nutrients cause a significant increase of long-term semi-pathological conditions such as oxidative stress, inflammation and dysbiosis, especially at intestinal level, which are the major cause of age-related chronic diseases and cancer^4^. Oxidative stress is due to an overproduced highly unstable and reactive chemical species, such as Reacting Oxygen Species (ROS) including radical forms (superoxide, O_2_^−^) and non-radical peroxide forms (H_2_O_2_). The production of these species is a physiological event, which occurs normally in the biochemical reactions of intestinal cells. Moderate concentrations of ROS are vital for intestinal homeostasis. They involve the regulation of intracellular signalling pathways, mediate the capacity of phagocytic tissue to kill bacteria and govern the interaction of the mucosa with the microbiome^5^. An imbalance of ROS causes overproduction of them and a consequent lipid peroxidation, DNA mutations, pro-inflammatory cytokine secretion and also other chronic complications, such as fibrosis, neoplasia and extra intestinal symptoms^6–9^. In addition, a modification of the redox balance can negatively compromise the health of microbiome altering its composition and activities with severe consequences for the long-term health of the individuals^10^. The growth and development of a robust gut microbiota is pivotal to maintain the intestinal homeostasis and human welfare as it can affect several homeostatic functions, including regulation of cellular growth, maintenance of barrier function and development of the immune system^11,12^. On the contrary, alterations of microbiota and gut dysbiosis can cause infections and diseases, especially when an overall decline in gut microbial diversity occurs^13^. Accordingly, there is an increased awareness about the key role played by the host microbiota in maintaining human health^14^.

The human organism has developed several mechanisms to counteract the damaging effects of oxidative stress, mediated by enzymatic and non-enzymatic antioxidants^15,16^. Natural antioxidants produced by the body or ingested with several food matrices or crops can reduce, or at least slow down, the oxidation reaction in the whole body and locally in the intestine. Long-term daily intake of natural antioxidants, in particular polyphenolic compounds, can prevent the onset of damages associated with oxidative stress, contributing to the maintenance of the human health and preserving the gut homeostasis^17,18^.

Grape is one of the largest fruit crops around the word, mostly cultivated to produce wine, and it is especially rich in bioactive components with promising properties in health promotion and disease management^19^. Epidemiological evidence has linked the consumption of grapes and wine with reduced risk of chronic diseases, including neurodegenerative and cardiovascular diseases^20,21^. In Sardinia, the daily and moderate consumption of red wine, which contains polyphenols, represents the secret of longevity^22^. Wine making leads to the production of a large volume of grape pomace, which represents an economic and environmental problem. The main chemical components of pomace are water (~ 50%), neutral polysaccharides (~ 20%), pectic substances (~ 20%), insoluble proanthocyanidins, lignin, structural proteins, and phenols (~ 15%)^23^. Among all, phenols (phenolic acids, flavanols, proanthocyanidins, flavonols, anthocyanins, and stilbenes)^24^ are the most interesting thanks to their antioxidant, anti-inflammatory, anti-neurodegenerative, anti-microbial, anti-cancer, and cardioprotective activities^25,26^. The high content of antioxidant compounds in grape pomace and seeds was previously demonstrated^27^. Unfortunately, at the moment, these bio-masses are considered a low value by-product or a waste, while their effective exploitation for the production of valuable nutraceutical products, should improve the welfare of human society, prevent the incorrect disposal of pomaces and increase the sustainability of the grape-wine chain thus promoting the circular economy^28^. Grape seeds mainly contain phenolics (up to 70% of total extractable compounds), omega-6 fatty acid, and vitamins^29^. The most abundant phenolic compounds are phenolic acids such as gallic acid, flavonoids such as monomeric catechin and epicatechin, and non-flavonoids such as stilbenes and procyanidins^27^. The last are dimers or, sometimes, trimers of flavanols, especially catechin and epicatechin^30^. All of these, except gallic acid, are low water soluble with low stability in biological fluids and as a consequence low bioavailable at the target sites, especially when orally administered^31,32^.

Previous studies disclosed that their delivery into suitable phospholipid vesicles can reduce these drawbacks improving their biological activities in the target sites^33,34^. Phospholipid vesicles ad hoc modified by using specific ingredients were able to improve the effectiveness of extracts and natural molecules in skin and intestine by improving their local bioavailability. In particular, penetration enhancer containing vesicles modified with water cosolvents (e.g. propylene glycol), polymer associate vesicles modified with eudragit, sodium hyaluronate or chitosan and nutriosomes improved with a dextrin, disclosed optimal carrier performances^34–36^.

In this study, a phytocomplex rich in antioxidants was obtained from the seeds of pomace of Cannonau cultivar. The resulted extract was loaded in phospholipid vesicles specifically tailored for intestinal delivery. In particular, transferosomes and hyalurosomes were selected because, in previous studies, they were able to improve the efficacy of different bioactives or phytocomlexes^37–39^. In addition, in this study, hyalutransfersomes were obtained by adding an edge activator to sodium hyaluronate immobilized vesicles (hyalurosomes).

The novelty of this work lies in the additional modification of these vesicles with Glucidex, a natural maltodextrin, thus obtaining glucidex-liposomes (used as reference), glucidex-transfersomes, glucidex-hyalurosomes and glucidex-hyalutransfersomes. Maltodextrin can act as structural component improving the stability of phospholipid bilayer and, at the same time, exert a functional role as prebiotic agent^40,41^. These new improved vesicles were used for the first time to maximize the effectiveness of the carrier for intestinal delivery.

The structure, morphology, size distribution, zeta potential, incorporation efficiency and antioxidant activity of vesicles were evaluated as well as their stability in acidic and basic environment. The biocompatibility of vesicles, their ability to protect the cells from oxidative stress and promote cell proliferation and migration, was assayed using intestinal epithelial cells (Caco-2). Finally, their prebiotic activity, as biofilm promoters, was tested in vitro using *Lactobacillus reuteri* as probiotic bacterium.

## Material and methods

### Materials

Lipoid S75 (S75), a mixture of soybean phospholipids (∼70% phosphatidylcholine, 9% phosphatidylethanolamine and 3% lysophosphatidylcholine), triglycerides and fatty acids, was kindly provided by AVG S.r.l. (Garbagnate Milanese, Milan, Italy), local supplier for Lipoid GmbH (Ludwigshafen, Germany). Sodium Hyaluronate was purchased from DSM Nutritional Products AG Branch Pentapharm (Switzerland). Tween 80, 1,1-diphenyl-2-picrylhydrazyl (DPPH), tetrazolium salt, 3-(4,5-dimethylthiazol-2-yl)-2,5-diphenyltetrazolium bromide (MTT) and all the other reagents were of analytical grade and were purchased from Sigma-Aldrich (Milano, Italy). Glucidex was kindly provided by Roquette Italia s.p.a (Cassano Spinola, Alessandria, Italy). Reagents and plastics for cell culture were purchased from Life Technologies Europe (Monza, Italia).

### Preparation of extract from pomace seeds

Fresh pomaces from Cannonau cultivar were kindly provided by Argiolas S.r.l. (Cagliari, Italy). Seeds were isolated from the pomace, frozen at − 18 °C and lyophilized to eliminate the water. Lyophilized seeds were then grinded for 5 min to obtain a fine powder with particles not larger than 5 μm, evaluated by using a manual multistage sieve. The obtained powder was vacuum packed until the extraction process (i.e. maceration) was performed. 100 g of grinded seeds were transferred in a beaker and ethanol 96° FU was added up 1,000 ml. The dispersion was kept under constant stirring for 48 h at room temperature (25 °C). The obtained extract was then filtered under vacuum and centrifuged at 2,500 rpm (Centrifuge Neya8 Basic, Sinergica Soluzioni S.r.l., Milan, Italy). The ethanol was eliminated by evaporation at 35 °C under vacuum and the remaining water was removed by lyophilization (Freeze-drier − 86 °C, Operon FDU8606, (Nuova Criotecnica Amcota, Rome, Italy) thus obtaining a dried and non-sticky power, which was immediately vacuum packed in dark glass container until its use^7,36^.

### HPLC analysis of extract

An ethanolic solution of the dried extract was prepared and analysed by using the HPLC–DAD method^42^. Extract compounds were detected and quantified by using an Agilent 1100 HPLC system (Agilent Technologies Italia S.p.A., Milan, Italy) consisting of a quaternary pump (G1311A), a rheodyne injector (G1313A), a thermostated column compartment (G1316A) and a degasser (G1322A), coupled with a DAD detector UV 6000 (Thermo Finnigan, Milan, Italy)^42^. To perform the analyses a C18 Kinetex column (5 μ, C18, 100 Å; Phenomenex, Torrance, CA, 5 USA) was used. The mobile phase was composed of acetonitrile and water with 0.22 M phosphoric acid. A linear elution mode with a flow rate of 0.4 ml/min was used: 0–20 min from 10 to 20% acetonitrile; 20–35 min from 20 to 30% acetonitrile; 35–70 min from 30 to 50% acetonitrile, then to 80% acetonitrile up to 120 min. Data detection was performed by using a DAD detector UV 6000 (Thermo Finnigan, Milan, Italy) by selecting a specific wavelength for each class of compound: 280 nm for phenolic acids and flavanols, 360 nm for flavonoids, and 520 nm for anthocyanins^37^.

The concentration of each analyte was quantified using external calibration curves and was expressed as mg/kg of raw material. The mean value of three injections of the same sample was reported.

### Vesicle preparation

Phospholipid S75 (120 mg/ml), glucidex (50 mg/ml) and seed extract (40 mg/ml) were weighed in glass vials and hydrated with water to obtain liposomes. Transfersomes were prepared with Tween 80, a single-chain surfactant, which can act as edge activators destabilizing the lipid bilayer and improving its deformability^43,44^. Hyalurosomes were obtained by dispersing sodium hyaluronate in water and using the dispersion as hydrating medium of vesicles. Hyalutransfersomes were prepared by adding Tween 80 to the phospholipid and hydrating them with the dispersion of sodium hyaluronate. Alternatively, the same vesicles were prepared by adding the glucidex to the phospholipid. The last is a natural maltodextrin with a dextrose equivalent of 17. To obtain a homogeneous and small vesicles, the resulted dispersions were sonicated (25 cycles, 5 s on and 5 s off) with a Soniprep 150 ultrasonic disintegrator (MSE Crowley, London, UK)^45,46^. Empty vesicles (extract-free) were also prepared and used as reference. The composition of the vesicles is reported in Table 1.

**Table 1:** Composition of seed extract loaded glu-liposomes, glu-transfersome, glu-hyalurosomes and glu-hyalutransfersomes.

	S75 (mg/ml)	Seeds extract (mg/ml)	Tween 80 (mg/ml)	Glucidex (mg/ml)	Sodium Hyaluronate (0.05% w/v) (ml)	Water (ml)
GLU-liposomes	120	40	–	50	–	1
GLU-transfersomes	120	40	5	50	–	1
GLU-hyalurosomes	120	40	–	50	0.5	0.5
GLU-hyalutransfersomes	120	40	5	50	0.5	0.5

The two last formulations were prepared with a dispersion of sodium hyaluronate (SH) 0.05% w/v.

### DPPH assay

The antioxidant activity of the extract in methanolic solution or loaded in vesicles was evaluated by the DPPH assay. The methanolic solution of the extract or the vesicle dispersions (40 µl) were diluted with a DPPH methanolic solution (40 µg/ml) and stored for 30 min in the dark at room temperature. The absorbance (ABS) was measured at 517 nm using a UV spectrophotometer. The concentration of samples required to obtain a 75% of antioxidant effect (EC75) was calculated and compared with that of quercetin.

### Vesicle characterization

Formation and morphology of vesicles were evaluated by cryo-TEM observation. A thin film of each sample was formed on a holey carbon grid and vitrified by plunging (kept at 100% humidity and room temperature) into ethane maintained at its melting point, using a Vitrobot (FEI Company, Eindhoven, The Netherlands). The vitreous films were transferred to a Tecnai F20 TEM (FEI Company), and the samples were observed in a low-dose mode. Images were acquired at 200 kV at a temperature of ~ − 173 °C, using a CCD Eagle camera (FEI Company)^47^.

Average diameter and polydispersity index of each sample (refractive index ~ 1.450) was evaluated by Photon Correlation Spectroscopy by using a Zetasizer nano (Malvern Instruments, Worcestershire, UK). The zeta potential was measured by means of M3-PALS method (phase analysis light scattering) using a Zetasizer nano. All the measurements were performed after dilution of the samples with water (refractive index 1.330)^48^.

To evaluate the amount of active ingredients incorporated into the vesicles, samples (2 ml) were purified by dialysis (Spectra/Por 172 membranes: 12–14 kDa 173 MW cut-off, 3 nm pore size; Spectrum Laboratories Inc., DG Breda, Netherlands) against water (2 L) for 2 h at room temperature (~ 25 °C), refreshing the water after 1 h to allow the complete removal of the non-entrapped molecules^49^. At the end of the purification process, the antioxidant activity of the samples, before and after dialysis, was measured by the DPPH assay (see “Vesicle preparation”), and the entrapment efficiency was calculated as a percentage of the antioxidant activity after dialysis versus that before dialysis.

### Vesicle stability under acidic and basic media at high ionic strength

An acid solution (pH 1.2) with high ionic strength was prepared by dissolving sodium chloride (20 mg/ml) in a solution of hydrochloric acid 1 M (6% v/v) in water. A solution at pH 7 with high ionic strength was prepared by dissolving Na_2_HPO_4_ (7.26 mg/ml), NaH_2_PO_4_ (3.56 mg/ml) and sodium chloride (17.54 mg/ml) in water.

In order to evaluate the ability of vesicles to maintain intact their structure under these harsh conditions, vesicle dispersions were diluted (1:100) with the prepared fluids thermostated at 37 °C and maintained for 2 h at pH 1.2 or for 6 h at pH 7^50,51^. Size, polydispersity index and zeta potential were measured at time 0 or after 2 or 6 h^36^.

The amount of extract retained inside the vesicles was measured as well, using a dissolution tester equipped with 6 stations (DT 720 Series—Erweka, distributed by EMME 3 SRL, Milan) that strictly complies with USP requirements. Vesicle dispersions were diluted (1:10) with the acid or basic solution, were transferred into polycarbonate dialysis tubes (Spectra/Por membranes: 12–14 kDa MW cut-off, 3 nm pore size; Spectrum Laboratories Inc., USA), putted in the baskets of the dissolution tester containing the release media (200 ml) and left under constant stirring at 37 °C for 2 h at pH 1.2 and 6 h at pH 7. The amount of extract released was calculated by measuring the antioxidant activity of the samples by means of DPPH test, at time 0, 2 h (pH 1.2) or 6 h (pH 7).

### Biocompatibility of extract in dispersion or loaded in vesicles

Caco-2 were used as a model of human intestinal cells (ATCC collection, USA). The cells were growth as monolayers in 75 cm^2^ flasks and incubated at 37 °C in a controlled atmosphere containing 5% CO_2_ and 100% humidity. The medium Dulbecco's Modified Eagle (DMEM) high glucose, containing l-glutamine, bovine foetal serum (10%), penicillin–streptomycin and fungizone was used to culture the cells^52^.

The toxic effect of the extract in aqueous dispersion or loaded in vesicles was evaluated by the MTT cell viability test. Cells (5 × 10^4^ cells/well) were seeded in 96 well plates. After 24 h, 25 μl of each formulation were diluted with the cell medium described above (enriched DMEM) to reach the desired concentration of the extract (0.2, 2, 20, 40 µg/ml). The resulted dispersions were added to the cell medium. After 48 h of incubation, cells were washed, the medium was removed and then 100 µl of MTT (0.5 mg/ml in PBS, final concentration) was added in each well and incubated for 3 h. The formed formazan crystals were solubilized with DMSO and the absorbance was read spectrophotometrically at 570 nm using a microplate reader (Multiskan EX, Thermo Fisher Scientific, Inc., Waltham, MA, USA)^36^. Results are reported as a percentage of viability of treated cells in comparison with that of untreated control cells (100% viability)^36^.

### Protective effect of grape extract in dispersion or loaded in vesicles against cell damages induced by hydrogen peroxide

To evaluate the ability of formulations to protect the CaCo-2 from oxidative stress, cells were seeded in 96-well plates (5 × 10^4^ cells/well) and incubated at 37 °C in a controlled atmosphere containing 5% CO_2_ and 100% humidity. After 24 h cells were exposed to hydrogen peroxide (25 μl; 1:40,000 dilution), and straightaway incubated for 4 h with 25 μl of the extract in dispersion or loaded in vesicles (2 μg/ml, final concentration of the extract). Cells treated only with hydrogen peroxide (without extract) were used as a positive control, whereas untreated cells were used as a negative control (100% vitality). After 4 h of incubation medium was removed, cells were washed with PBS and the number of live cells was determined by the MTT colorimetric assay, as reported above (“Biocompatibility of extract in dispersion or loaded in vesicles”).

### In vitro scratch assay

The ability of the formulations to stimulate the migration and proliferation of Caco-2 cells was evaluated by using the scratch assay test^39,53^. Cells were seeded in 6-well plates and kept in culture until the confluence was reached. Subsequently, a thin wound was generated on the cell monolayer using a sterile plastic tip. Cell fragments were removed by gently washing each well with the medium preheated at 37 °C. Immediately after the generation of the wound (time 0), cells were treated with the extract in dispersion or loaded in vesicles (2 μg/ml of extract) and incubated up to 96 h. Untreated cells were used as control. At each time point (0, 48 and 96 h) cells were observed using an optical microscope to monitor cell proliferation and migration and wound closure (10 × objective)^39,53^.

### Study of *Lactobacillus reuteri* biofilm formation

The ability of formulations to increase the probiotic strain biofilm was evaluated in vitro following the crystal violet staining protocol. Briefly 1 × 10^6^ cells/mL of *Lactobacillus reuteri* DSM 17,938 (German Collection of Microorganisms and Cell Cultures) were inoculated into 96-well microplate containing Schaedler Broth (Microbiol, Uta, Cagliari) and treated with different dilutions of extract loaded vesicles. After 24 h at 37 °C with 5% CO_2_ the medium was discarded, wells were gently washed three times with NaCl solution (0.9%) and crystal violet (0.1 ml, 0.1%) solution was added. After 10 min the dye was discarded, the well washed three times with NaCl solution, dried an air at 25 °C for 15 min and acetic acid (0.3 ml, 30%) was added to each well. The absorbance of each well was read with a microplate reader at 450 nm (SLT-Spectra II, SLT Instruments, Germany)^11,54^. The experiments were performed in triplicate.

### Statistical analysis of data

The results were expressed as mean value ± standard deviation. Statistically significant differences among samples were determined using variance analysis. The t-test was used to substantiate a significant difference between the means of two specific groups. The statistical analysis was performed by using the Excel software package (Microsoft Corp, Redmond, USA) equipped with a tool for statistical analysis. The minimum level of significance chosen was P < 0.05.

## Results

### Extraction of seed phytocomplex and identification of its components

The grape seeds were separated from the pomace of Cannonau cultivar and grinded before the extraction to improve its specific surface and facilitate the extraction yield. The main components of seed extract, as previously detected by HPLC–DAD, were: epicatechin, catechin, epicatechin gallate, procyanidin B1 and B2, gallic acid, quercetin and malvidin-3-glucoside (Table 2). Catechin and epicatechin were the most abundant, 88 and 170 mg/kg, respectively^42^.

**Table 2:** Amounts of bioactive molecules (mg) contained in the extract (kg) obtained from grape seeds.

Components	Concentration mg/kg
*Hydroxybenzoic acid*	
Gallic acid	35.60 ± 3.17
*Flavan 3-ols*	
(+) Catechin	88.40 ± 2.66
(−) Epicatechin	170.47 ± 4.83
(−) Epicatechin gallate	61.24 ± 2.03
(−) Epigallocatechin gallate	nd
Procyanidin B1	52.82 ± 1.84
Procyanidin B2	41.57 ± 1.52
Others procyanidins^a^	253.15 ± 6.80
*Flavonols*	
Myricetin	5.28 ± 0.32
Quercetin	31.57 ± 1.17
Quercetin 3-glucoside	7.54 ± 0.32
Quercitrin	4.76 ± 0.15
*Anthocyanins*	
Petunidin 3-glucoside^e^	0.58 ± 0.00
Peonidin 3-glucoside	1.37 ± 1.17
Malvidin 3-glucoside	8.70 ± 0.33
Delphinidin-3-acetilglucoside^b^	0.93 ± 0.00
Malvidin-3-acetilglucoside^c^	Traces
Cyanidin-3-p-coumaroilglucoside^d^	Traces
Petunidin-3-p-coumaroilglucoside^e^	Traces
Peonidin-3-p-coumaroilglucoside^e^	Traces
Malvidin-3-p-coumaroilglucoside^c^	Traces

Mean values ± standard deviations are reported (n = 3).

^a^Expressed as equivalent of Procyanidin B1.

^b^Expressed as equivalent of Delphinidin-3-glucoside.

^c^Expressed as equivalent of malvidin 3-glucoside.

^d^Expressed as equivalent of cyaniding 3-glucoside.

^e^Expressed as equivalent of peonidin 3-glucoside.

The antioxidant activity of the seed extract, measured by means of the DPPH assay, was ~ 9% of that of pure quercetin measured at the same concentration and conditions. Quercetin was used as a reference because it is one of the main components of the extract and one of the most powerful natural antioxidants.

### Preparation and characterization of seed extract loaded vesicles

To improve the stability and bioavailability of the phytocomplex extracted from the grape seeds, phospholipid vesicles specifically tailored for intestinal delivery such as glucidex-transfersomes, glucidex-hyalurosomes and glucidex-hyalutransfersomes, have been formulated and tested. Corresponding glucidex-liposomes, prepared with the same amount of phospholipid, glucidex and extract, were prepared and used as reference.

**Figure 1:**
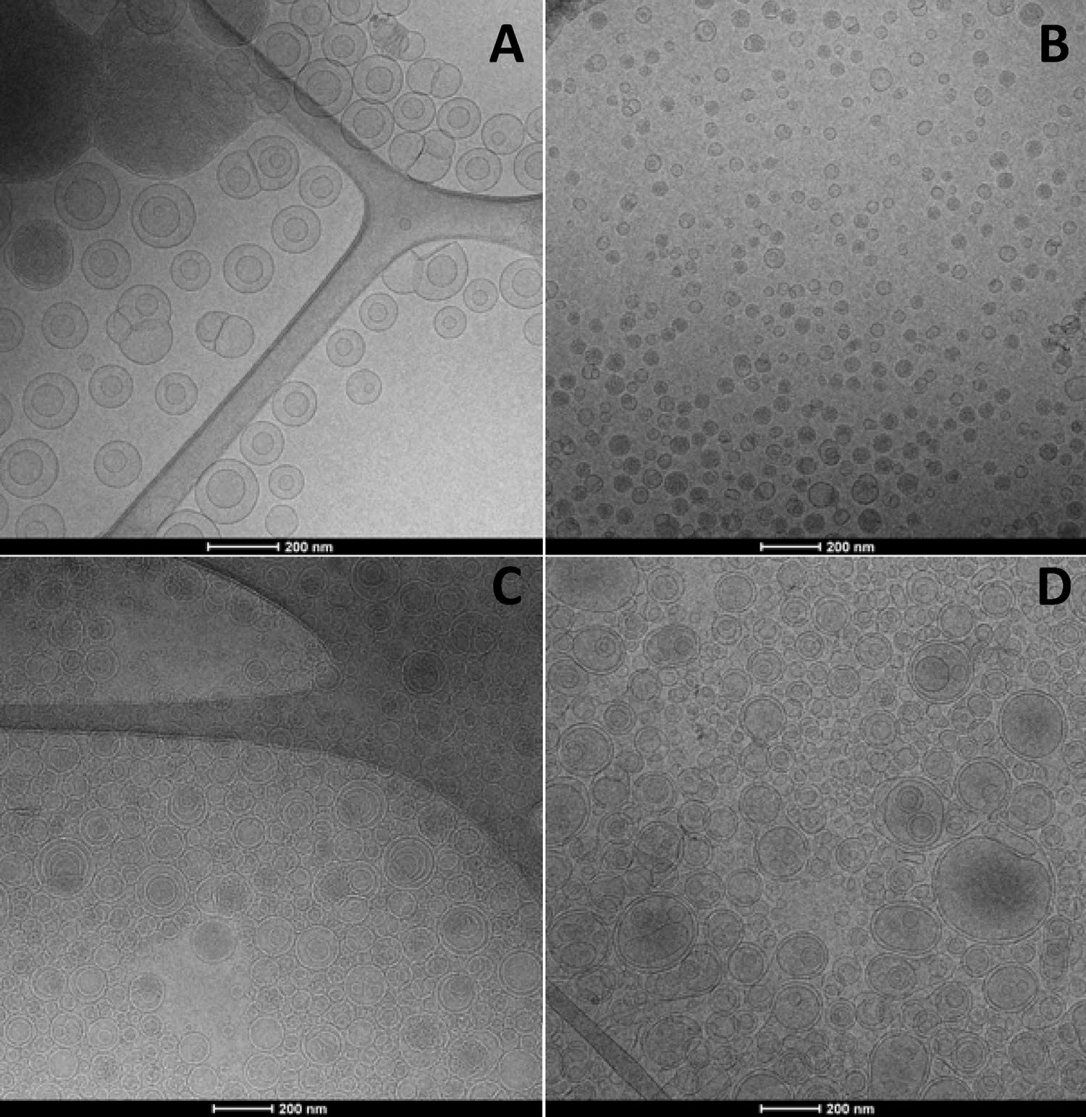
Representative cryo-TEM images of glucidex-liposomes (**A**), glucidex-transfersomes (**B**), glucidex-hyalurosomes (**C**) and glucidex-hyalutransfersomes (**D**). Magnification × 100,000.

Glucidex-liposomes and glucidex-transfersomes were mainly unilamellar vesicles, while the addition of sodium hyaluronate led to the formation of oligolamellar vesicles, as detected by cryo-TEM analyses (Fig. 1).

The physico-chemical properties of vesicles were evaluated by measuring the mean diameter, polydispersity index and zeta potential. Empty vesicles were prepared and characterized as well, to evaluate the effect of the extract loading on vesicle assembling. Empty glucidex-liposomes (~ 79 nm) and glucidex-hyalurosomes (94 nm) were the smallest (P < 0.05 between the two samples). The addition of Tween 80 in glucidex-transfersomes and glucidex-hyalutransfersomes caused an increase of the mean diameter up to ~ 113 nm (P > 0.05 between the two samples). The loading of the extract allowed a strong increase of the mean diameter of all the vesicles (~ 151 nm, P < 0.05 versus empty vesicles) without significant differences between the four formulations (P > 0.05). This result indicates the effective intercalation of the components of phytocomplex within the bilayer, leading to a modification of its assembling and a reduction of the curvature radius^48^. All formulations were homogeneously dispersed, in particular, the polydispersity index of glucidex-transfersomes, glucidex-hyalurosomes and glucidex-hyalutransfersomes was ≤ 0.27 denoting a monodispersed system (Table 3).

**Table 3:** Mean diameter (MD), polydispersity index (PI), zeta potential (ZP) and entrapment efficiency (EE) of empty and seeds extract loaded glucidex-liposomes, glucidex-transfersomes, glucidex-hyalurosomes and glucidex-hyalutransfersomes.

Samples	MD (nm)	PI	ZP (mV)	EE (%)
Empty glu-liposomes	79 ± 12^§^	0.27	− 77 ± 8	–
Empty glu-transfersomes	109 ± 13^#^	0.24	− 72 ± 13	–
Empty glu-hyalurosomes	94 ± 5°	0.23	− 66 ± 3	–
Empty glu-hyalutransfersomes	117 ± 14^#^	0.30	− 66 ± 2	–
Extract glu-liposomes	147 ± 29*	0.31	− 74 ± 7	90 ± 9
Extract glu-transfersomes	149 ± 15*	0.27	− 81 ± 5	96 ± 6
Extract glu-hyalurosomes	152 ± 17*	0.27	− 76 ± 7	97 ± 8
Extract glu-hyalutransfersomes	155 ± 26*	0.25	− 80 ± 12	96 ± 8

Mean values ± standard deviations were reported (n = 6). Each symbol (*, ^§^, °, ^#^) indicates a different value (P < 0.05).

Zeta potential was strongly negative for all the vesicles which is predictive of a good stability of the dispersions, due to the repulsive forces among particles, which can avoid aggregation and fusion phenomena.

The grape seed extract was incorporated in high amount into the vesicles without significant differences among samples. Indeed, the entrapment efficiency was greater than 90% for all the formulations (Table 3).

### Antioxidant activity of seed extract in dispersion or loaded in vesicles

The antioxidant activity of the extract loaded in vesicles was measured by using the DPPH test and compared with that of a methanolic solution of the extract and that of the quercetin. The antioxidant activity was evaluated taking into account that the main components of the extract such as gallic acid, catechins, quercetin, malvidins and procyanidins, can scavenge the free radicals and prevent peroxidation. The IC75 of the quercetin was 9.76 mg/ml, that of the grape extract solution was 0.83 mg/ml and that of all the vesicle dispersions was 0.82 mg/ml. The antioxidant activity of the extract in solution or loaded in vesicles was the same confirming that the loading did not modify the antioxidant power of the extract.

### Stability studies at 37 °C in solution at pH 1.2 and pH 7 with high ionic strength

**Figure 2:**
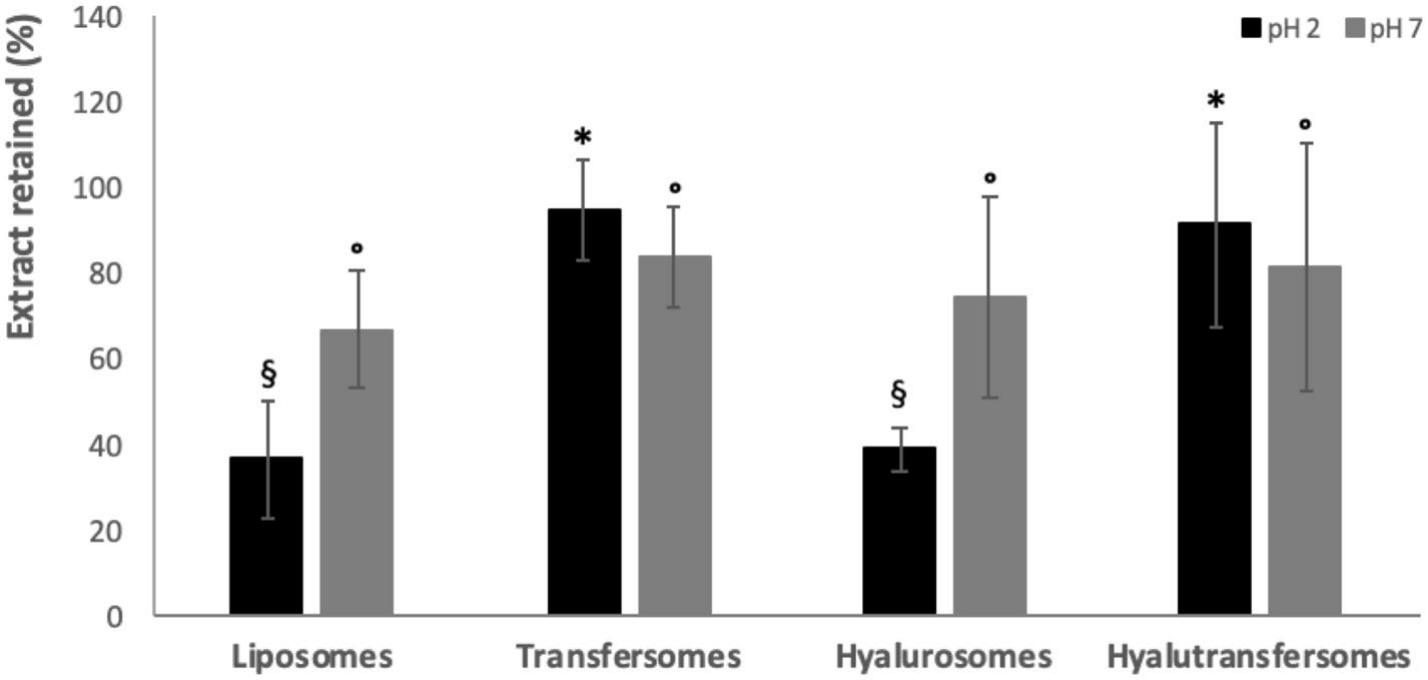
Amount (%) of extract retained into the vesicles after incubation at pH 1.2 (2 h) and pH 7 (6 h). The mean values ± standard deviations (error bars) are reported (n = 3). Each symbol (*, ^§^, °) indicates a different value (P < 0.05).

Oral administration involves the passage of formulations through the gastro-intestinal tract, characterized by acidic or neutral media with high ionic strength. These harsh conditions, especially in the stomach (pH 1.2) can destabilize or destruct the vesicles. The stability of extract loaded vesicles was tested by measuring their mean diameter, polydispersity index, zeta potential and the amount of phytocomplex retained (Table 4 and Fig. 2) by diluting them with media mimicking the gastro-intestinal environment. After dilution and incubation at 37 °C for 2 h at pH 1.2, the size and polydispersity index of glucidex-liposomes and glucidex-hyalurosomes strongly increased reaching ~ 3,000 nm and 0.8 of polydisperison. This behaviour can be due to the fusion and aggregation of some vesicles forming larger ones. The zeta potential was reversed to slightly positive values due to the presence of ionized protons in solution, as previously reported^55,56^. The zeta potential values of the vesicles in dispersion is closely related to the ionic strength of external medium^57^ and each variation in the ionic strength of the medium affects the disposition of the charge on vesicle surface^58^ and the zeta potential value. At acidic pH, the increased number of protons in the medium interacts with the amphipathic phosphatidylcholine molecules surrounding the vesicles and reversing their double electron layer to positive values.

**Table 4:** Mean diameter (MD), polydispersity index (PI), zeta potential (ZP) of grape seed extract loaded vesicles diluted with a solution at pH 1.2 or pH 7 and high ionic strength, kept at 37 °C for 2 or 6 h, respectively.

	pH	MD (nm)	PI	ZP (mV)
Glu-liposomes	pH 1.2 (t_2h_)	2,343 ± 297°	0.86^#^	9 ± 1
	pH 7 (t_6h_)	230 ± 27^#^	0.31*	− 7 ± 1
Glu-transfersomes	pH 1.2 (t_2h_)	354 ± 68*	0.32*	7 ± 1*
	pH 7 (t_6h_)	186 ± 26^$^	0.19^+^	− 6 ± 1
Glu-hyalurosomes	pH 1.2 (t_2h_)	3,629 ± 380^§^	0.95^#^	8 ± 1
	pH 7 (t_6h_)	255 ± 33^#^	0.30*	− 6 ± 1
Glu-hyalutransfersomes	pH 1.2 (t_2h_)	387 ± 54*	0.37*	8 ± 3
	pH 7 (t_6h_)	169 ± 2^$^	0.13^+^	− 6 ± 2

The mean values ± standard deviations are reported (n = 3). Each symbol (*, ^§^, °, ^#^, ^$^) indicates a different value (P < 0.05).

Surprisingly, the mean diameter and polydispersity index of vesicles containing Tween 80 (glucidex-transfersomes and glucidex-hyalutransfersomes) underwent only a slightly increase of their size (~ 378 nm and 0.34) at pH 1.2. The zeta potential was reversed into positive values as detected for liposomes and hyalurosomes. Despite, the modified zeta potential, glucidex-transfersomes and glucidex-hyalutransfersomes did not change their size (~ 378 nm and 0.34) disclosing a good stability, which seem to be related to the presence of the edge activator Tween 80. It makes the vesicles more deformable minimizing the structural changes caused by the increased ionic species and protons.

After dilution and incubation at 37 °C for 6 h at pH 7, glucidex-liposomes and glucidex-hyalurosomes were more stable than at pH 1.2 and only underwent a slight increase of mean diamemeter (~ 260 nm). Differently, the mean diameter of transfersomes and hyalutransfersomes remained almost unchanged (~ 178 nm), and the polydispersity index slightly decreased. For the four formulations the zeta potential increased to slightly negative values (to ~ -6 mV). The stability results at pH 1.2 and 7, disclosed that the presence of the edge activator (Tween 80) in transfersomes and hyalutransfersomes probably made the vesicle bilayer more elastic and resistant to acid conditions ensuring vesicle stabilization and avoiding breaking or fusion in larger ones.

Indeed, the leakage of extract from these vesicles, after 2 h of incubation at pH 1.2, was ~ 10% being the amount of the retained extract ~ 90% (Fig. 2). According to the strong increase of mean diameter and polydispersity index of vesicles, the amount of extract retained in glucidex-liposomes and glucidex-hyalurosomes was ~ 37%, probably because break and fusion of some vesicles occurred leading the leakage of extract. After incubation at pH 7 all the vesicles retained a high amount of phytocomplex, ~ 70%, without significant differences between the formulations.

### In vitro biocompatibility by using CaCo-2 cells

**Figure 3:**
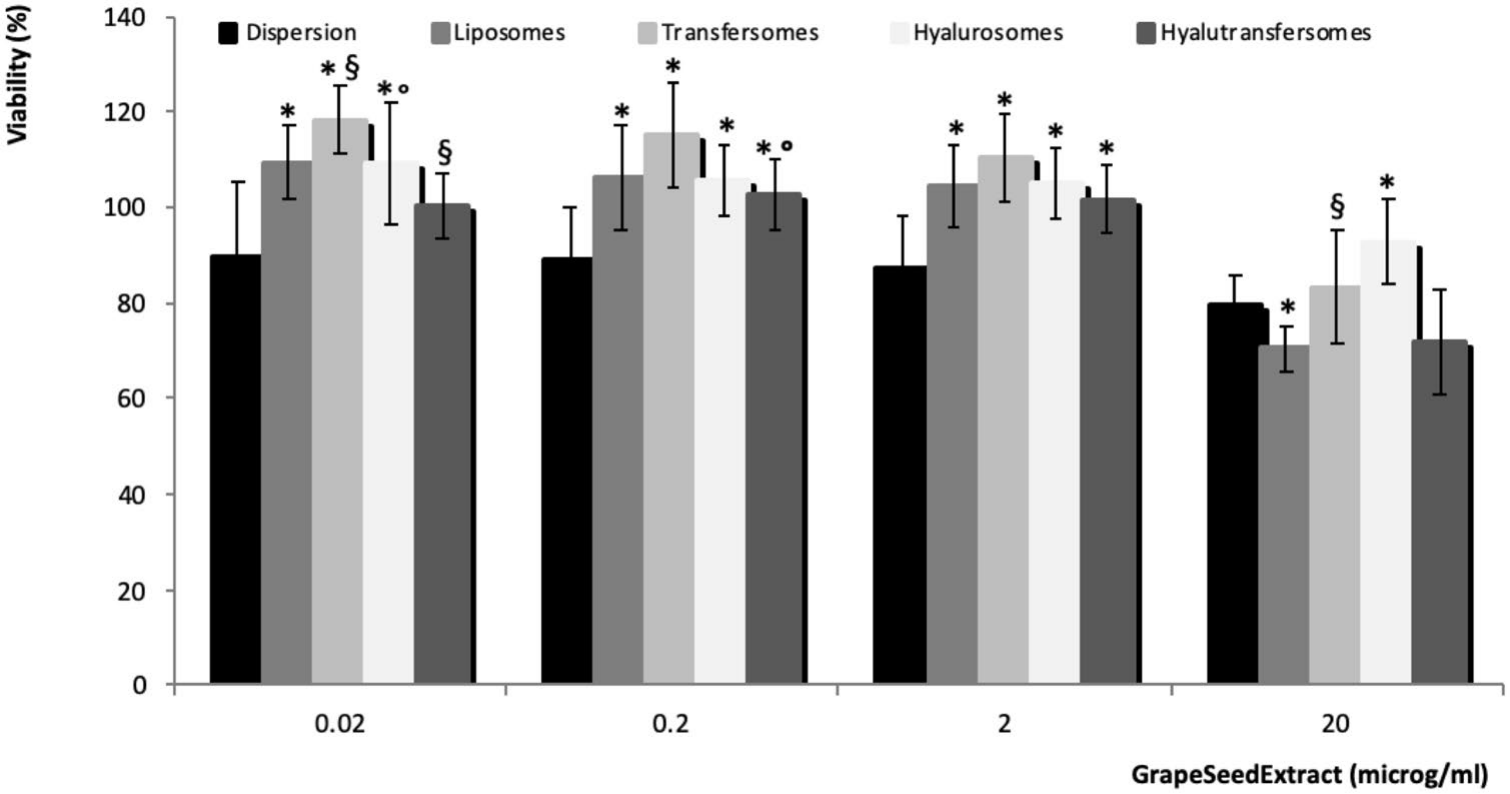
Viability of Caco-2 cells incubated for 48 h with the extract in aqueous dispersion or loaded in vesicles. The mean values ± standard deviation (error bars) have been reported. The symbol * indicates values that were statistically different from the extract dispersion; the symbol § indicates values that were statistically different from liposomes and the symbol ° indicates values that were statistically different from transfersomes (P < 0.05).

The biocompatibility of extract loaded vesicles was evaluated using Caco-2 cells which represent the most widely used model of human intestinal cells. To this propose, the aqueous dispersion of seed extract or extract loaded vesicles were diluted in cell medium to reach 20, 2, 0.2, 0.002 μg/ml of extract and used as culture medium of Caco-2. After 48 h of incubation, cell viability was measured by using the MTT test (Fig. 3). The viability of cells treated with the aqueous dispersion of the extract was ~ 88% irrespective to the used concentration. When the cells were treated with the extract loaded in vesicles, irrespective to their composition, the viability remained ~ 88% using the higher concentration 20 μg/ml and increased over 100% with the lower concentrations. The obtained findings underline that the extract loaded vesicles at concentrations lower than 2 μg/ml of extract did not show any toxic effect but even can exert a proliferative effect.

### Protective effect of grape seed extract against oxidative stress induced in Caco-2 cells

**Figure 4:**
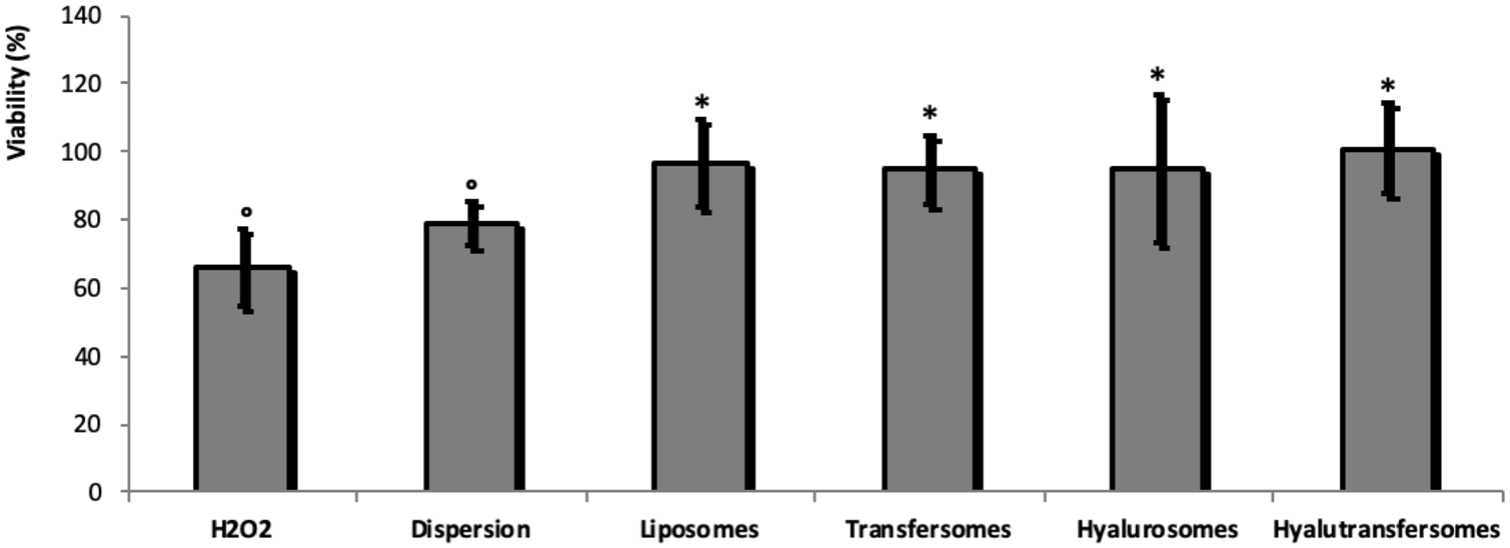
Viability of Caco-2 cells stressed with hydrogen peroxide and incubated for 4 h with the seed extract (2 μg/ml) in water or loaded in vesicles. Mean values ± standard deviations (error bars) are reported (n = 8). Each symbol (*, °) indicates a different value (P < 0.05).

To evaluate the protective effect of extract loaded vesicles against oxidative stress, Caco-2 cells were stressed with hydrogen peroxide and simultaneously treated with the extract in dispersion or loaded in vesicles. The higher non-toxic concentration selected in the previous study was used (2 μg/ml of extract). Hydrogen peroxide was used as stressing agent as it is considered the main cause of cell death between active oxygen species. The cytotoxic effect was assessed measuring the cell viability by the MTT test^55, 56^. The treatment with hydrogen peroxide caused a significant reduction of cell viability (~ 66%) and its damages was partially reduced by simultaneously treating the cells with the dispersion of the raw extract in water, as the cell viability slightly increased up to ~ 79% (Fig. 4).

When the cells were treated with the extract loaded into vesicles, irrespective to their composition, the viability was always ~ 100%, probably because the vesicles were able to promote the antioxidant activity of phytocomplex and its cell internalization^59^.

### Effect of grape seed extract on proliferation and migration of Caco-2

**Figure 5:**
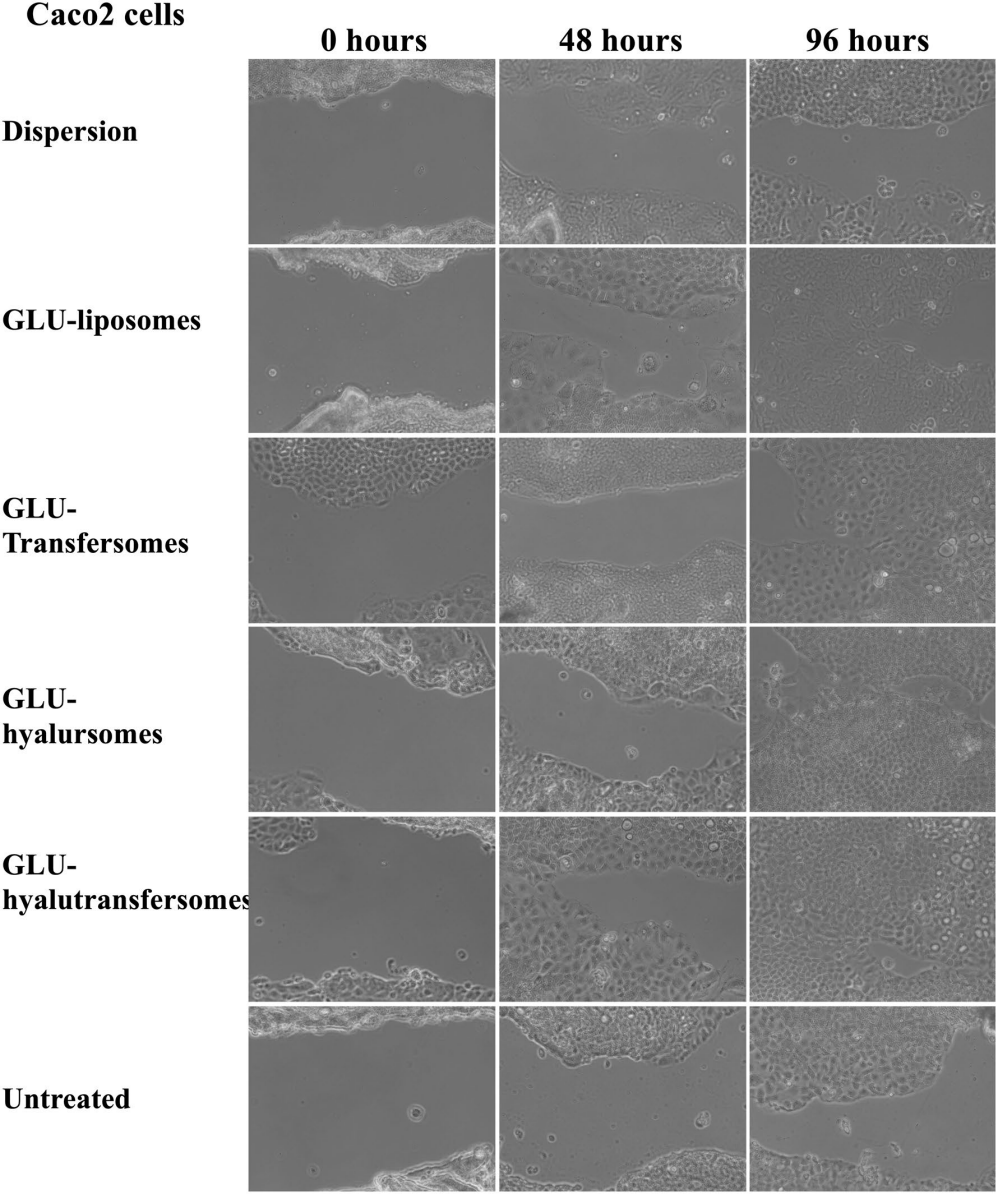
Representative images of a scratch in a monolayer of Caco-2 cells untreated or treated with the extract in water dispersion or loaded in vesicles.

The effect of seed extract loaded vesicles on the stimulation of cell proliferation and migration was evaluated in vitro by means of the scratch assay method^35^. The free extract dispersed in water at the same concentration was used as reference (Fig. 5). The untreated cells partially proliferated and migrated and at 96 h the lesions (scratches) were reduced but still visible. The treatment with the free extract in water slightly improved the lesion closure while the treatment with the extract loaded vesicles stimulated in a better extent, the migration and proliferation of the cells in the region of the lesion. Indeed, at 96 h, only the cells treated with extract loaded vesicles allowed the almost complete closure of the lesion, in particular the hyalurosomes and hyalutransfersomes seemed to be the most effective which ensured the almost complete closure of the intestinal cell lesion.

### Effect of seed extracts on Lactobacillus reuteri biofilm

**Figure 6:**
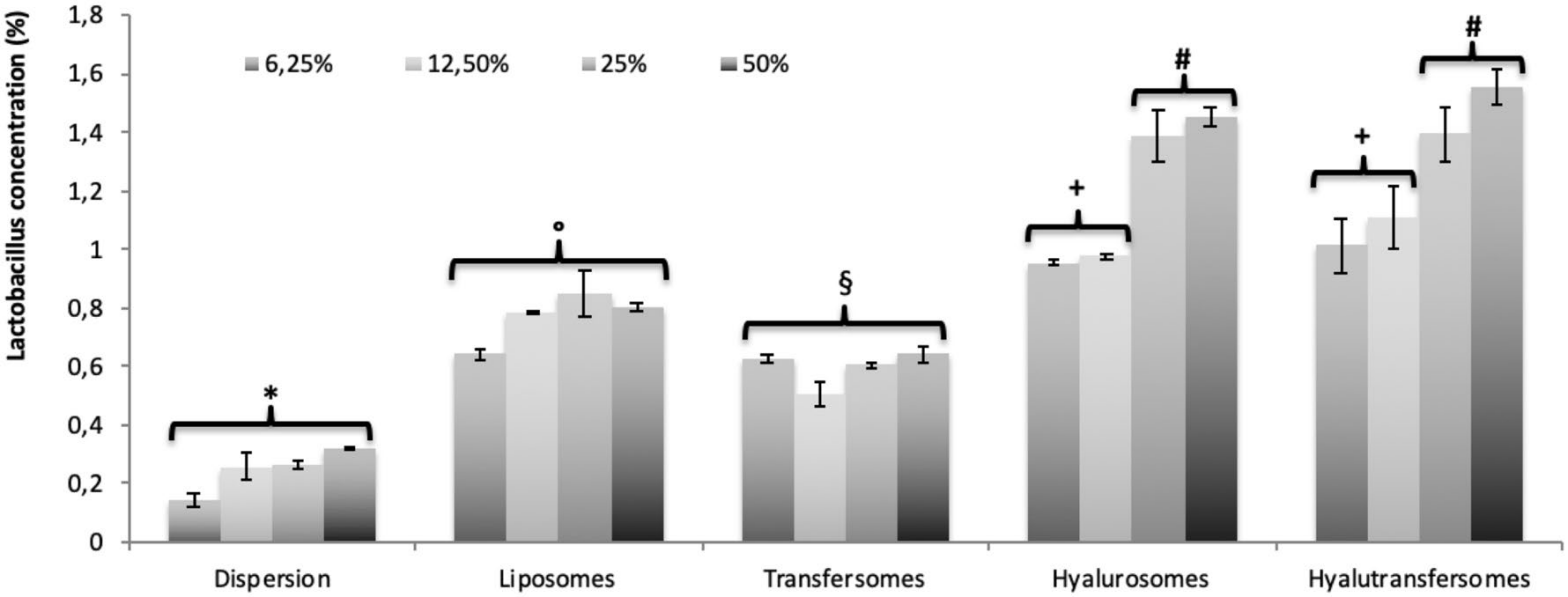
Effect of the seed extract in water dispersion or loaded in vesicles, on the biofilm in vitro of the probiotic strain *Lactobacillus reuteri*. Mean values ± standard deviations (error bars) are reported (n = 8). Each symbol (*, ^§^, °, ^+^ , ^#^) indicates a different value (P < 0.05).

The ability of extract loaded vesicles to interact with the formation process of *L. reuteri* biofilm was evaluated. The results suggest a positive influence of all the formulations on the biofilm mass (Fig. 6). The aqueous dispersion of the extract addressed a concentration of biofilm ~ 0.2%, irrespective to the extract concentration. Using glucidex-liposomes and glucidex-transfersomes the mass concentration increased up to ~ 0.6%, irrespective to the concentration. When glucidex-hyalurosomes and glucidex-hyalutransferosomes were tested the resulted biofilm was affected by the concentration of the extract into the vesicles: using the highest concentration the amount of biofilm increased up to ~ 1.5%, resulting sevenfold higher than that provided by the extract in dispersion. The positive effect of vesicle formulations should be related to the presence of the maltodextrin especially when associate with sodium hyaluronate.

## Discussion

In the last years the inappropriate heating habits combined with stress, physical inactivity and lack of sleep led to a significant increase of long-term semi-pathological conditions^4^. Recent evidence suggests the key role played by natural antioxidant, anti-inflammatory or prebiotic molecules in preventing or counteracting these problems and contributing to the maintenance of the human health^34,60,61^.

Grape is one of the largest fruit crops around the word, that contains a combination of resveratrol, quercetin, malvidin and other phytoactives, such as several catechins, anthocyanins, and flavonols^62^. This fruit is mainly used to obtain wine, which is connected with the production of a large amount of wastewater and organic waste that must be treated adequately to avoid the contamination of the areas of production^63^. However, these wastes, especially the pomace are still rich in bioactives of grape because only a small part of them pass in the wine^64,65^. Pomace is formed by the dried skin, stalks and seeds of grape. The last represent a suitable matrix containing fibres, oil, proteins, and 7% of complex phenols such as tannins, phenolic acids, anthocyanins, flavonoids, and proanthocyanidin complexes^66^.

In this study, the phytocomplex extracted from seeds of Cannonau pomace was obtained by maceration in ethanol. Ethanol was selected based on its complete biocompatibility and solubilizing-power. As previous reported^67^, aqueous solutions of ethanol were better than ethanol alone for the extraction of total phenols from grape seeds. The main components previously found in the ethanolic extract were active polyphenols such as epicatechin, catechin, epicatechin gallate, different procyanidins, gallic acid, quercetin and malvidin-3-glucoside^42^. Considering the potential antioxidant and prebiotic activity of these molecules and their low bioavailability, the extract was loaded in phospholipid vesicles specifically tailored for intestinal delivery^68^.

The formulations were designed to improve the intestinal bioavailability of bioactives ensuring an effective protection against oxidative stress and dysbiosis. Indeed, recent studies underlined that the incorporation of phytocomplexes in ad hoc modified phospholipid vesicles, can improve their local efficacy in vivo^69^. Recently, De Leo et al.^70^ incorporated curcumin in eudragit-coated liposomes to create a gastro-resistant carrier, able to protect the payload and deliver it in the colon. Hasibi et al^71^ formulated transfersomes containing Tween 80 or Span 60 and confirmed their ability to slowly release taxifolin at intestinal level without evaluating their stability at acidic pH.

In the light of this, liposomes were modified by selected additives (Tween 80, sodium hyaluronate and glucidex) to ameliorate the delivery performances of resulted vesicles: glucidex-transfersomes, glucidex-hyalurosomes and glucidex-hyalutransfersomes^72,73^. Tween 80 was added to make the vesicles (transfersomes) more flexibles and elastic^74^. Indeed, it acts as an edge activator, which alters the phospholipid assembling allowing its deformation. These peculiar characteristics of the transferosomes ensure their optimal performances as skin delivery systems. In this study, we underline the important role played by the Tween 80 in improving the ability of vesicles to keep intact their structure at acidic pH. This ability seems to be related to the increased deformability, which avoid vesicle destruction and the consequent leakage of the payload. Sodium hyaluronate is a naturally occurring polysaccharide with restoration and the healing properties^75^. It is widely used in the design of new delivery systems due to its biofunctionality associated to bioadhesive and viscoelastic properties^76,77^. It was also used to formulate phospholipid vesicles (hyalurosomes) for skin application^78^.

Glucidex, derived from the partial hydrolysis of starch and with a dextrose equivalent of 17, has been selected to ameliorate the gastric-resistance of vesicles because a previous study demonstrated that maltodextrins with a dextrose equivalent lower than 20 are stable under acidic conditions^79^. Glucidex has prebiotic effect and in oral formulations inhibits gut colonization by pathogens and promote the formation of the beneficial biofilm, thus exerting a protective effect against acute and chronic gut disorders^80,81^. It was previously demonstrated that the combination of phospholipid vesicles with polymers (i.e. hyaluronic acid) or fibres, improved the local bioavailability of natural bioactives after oral administration^35,82,83^.

In the present study, transfersomes were specifically modified with glucidex and sodium hyaluronate. Tween 80 and glucidex with phospholipid vesicles, (with or without sodium hyaluronate) seem to create a fruitful association. Accordingly, Barbosa et al. demonstrated that the combination of maltodextrins and Tween 80 in microcapsules ensured the incorporation of high amount of bixin and its protection against processing and storage conditions (high temperature, light and oxygen)^84^. Results suggest a positive interaction of Tween 80 and maltodextrin capable of stabilizing the carriers. In addition, using glucidex-hyalutransfersomes, sodium hyaluronate can exert its beneficial properties in the gut. Indeed, it is known that sodium hyaluronate promotes the proliferation and regeneration of gastrointestinal epithelia in injury models, as well as contributes to the regulation of normal intestinal and colonic growth^85^.

Vesicles were highly biocompatible and effectively counteracted the damaging effects induced by hydrogen peroxide in Caco-2 cells exerting a protective and preventive effect on gut epithelia. This is an important result as the oxidative stress can play an important role in mediating specific cell responses and expression of genes involved in degenerative pathophysiologic states, such as inflammation and cancer^86^. In addition, glucidex-hyalutransferosomes were able to promote in a better extent the migration and proliferation of the epithelial intestinal cells favouring the re-epithelialization of impaired or damaged tissue, which if untreated can undergo persistent epithelial defects with serious medical implications^87^.

Moreover, previous studies demonstrated the ability of grape polyphenols to modify the gut morphology and intestinal microflora, increasing the biodiversity degree of intestinal bacteria in broiler chicks^88,89^, acting as prebiotics that positively affect the host by stimulating both growth and activity of beneficial bacteria of the intestinal microbiota^90^. These beneficial properties of the grape pomace extract are even potentiated by its loading into glucidex-hyalutransferosomes, as they effectively promoted the proliferation of *Lactobacillus* biofilm contributing at counteracting the dysbiosis of the microbiota often associated with oxidative stress and inflammatory conditions.

## Conclusions

In this work the extract obtained from Cannonau grape seeds rich in antioxidants was successfully loaded in glucidex-transfersomes, glucidex-hyalurosomes and glucidex-hyalutransfersomes. Seed grape extract loaded glucidex-hyalutransferosomes, obtained by loading the extract in phospholipid containing Tween 80, glucidex, and sodium hyaluronate seem to be a promising system for the protection and care of human intestine. This kind of products can gain large interest in the modern society, due the currently attention to the natural and safe products with health-promoting properties. Moreover, the development of this natural, environmentally friendly, safe and affordable formulation based on grape seed extract, soy lecithin, Tween 80, sodium hyaluronate and maltodextrin can be achieved with limited economic and environmental costs thus contributing to improve the circular economy and achieve the goal of green chemistry innovation. The grape pomace will be recycled to manufacture food supplements aimed at maintaining the human health.
